# Association between the consumption of ultra-processed foods and the incidence of peptic ulcer disease in the SUN project: a Spanish prospective cohort study

**DOI:** 10.1007/s00394-024-03439-2

**Published:** 2024-05-29

**Authors:** Alessandro Leone, Carmen De la Fuente-Arrillaga, Mariano Valdés Mas, Carmen Sayon-Orea, Francesca Menichetti, Miguel Angel Martínez-Gonzalez, Maira Bes-Rastrollo

**Affiliations:** 1 International Center for the Assessment of Nutritional Status and The Development of Dietary Intervention Strategies (ICANS-DIS), Department of Food, Environmental and Nutritional Sciences (DeFENS), University of Milan, Milan, Italy; 2 Clinical Nutrition Unit, Department of Endocrine and Metabolic Medicine, IRCCS Istituto Auxologico Italiano, Milan, 20100 Italy; 3 Department of Preventive Medicine and Public Health, School of Medicine, University of Navarra, Pamplona, Spain; 4 CIBERobn, Instituto de Salud Carlos III, Madrid, Spain; 5 Navarra Institute for Health Research (IdiSNA), Pamplona, Spain; 6 Digestive Department, University of Navarra Clinic, Pamplona, Spain; 7 Navarra Institute of Public Health, Pamplona, Spain

**Keywords:** Ultra-processed food, Peptic ulcer disease, NOVA food classification system, Gastrointestinal disorders

## Abstract

**Purpose:**

Consumption of ultra-processed foods (UPF) has increased despite potential adverse health effects. Recent studies showed an association between UPF consumption and some gastrointestinal disorders. We evaluated the association between UPF consumption and peptic ulcer disease (PUD) in a large Spanish cohort.

**Methods:**

We conducted a prospective analysis of 18,066 participants in the SUN cohort, followed every two years. UPF was assessed at baseline and 10 years after. Cases of PUD were identified among participants reporting a physician-made diagnosis of PUD during follow-ups. Cases were only partially validated against medical records. Cox regression was used to assess the association between baseline UPF consumption and PUD risk. Based on previous findings and biological plausibility, socio-demographic and lifestyle variables, BMI, energy intake, Helicobacter pylori infection, gastrointestinal disorders, aspirin and analgesic use, and alcohol and coffee consumption were included as confounders.We fitted GEE with repeated dietary measurements at baseline and after 10 years of follow-up. Vanderweele’s proposed E value was calculated to assess the sensitivity of observed associations to uncontrolled confounding.

**Results:**

During a median follow-up of 12.2 years, we recorded 322 new PUD cases (1.56 cases/1000 person-years). Participants in the highest baseline tertile of UPF consumption had an increased PUD risk compared to participants in the lowest tertile (HR = 1.52, 95% CI: 1.15, 2.00, P_trend_=0.002). The E-values for the point estimate supported the observed association. The OR using repeated measurements of UPF intake was 1.39 (95% CI: 1.03, 1.87) when comparing extreme tertiles.

**Conclusion:**

The consumption of UPF is associated with an increased PUD risk.

**Supplementary Information:**

The online version contains supplementary material available at 10.1007/s00394-024-03439-2.

## Introduction

Ultra-processed foods (UPF) are industrial formulations including, besides salt, sugar, oils, and fats, food-derived substances and additives, the aim of which is to make these products attractive, extremely palatable and convenient (ready-to-eat and with a long shelf life) [1]. Their consumption has increased dramatically in recent decades, so much so that nowadays they contribute 50% or more of daily calorie intake in some Western countries [2–4]. It has been reported that a diet rich in UPF is nutritionally unbalanced [5], and several prospective cohorts have repeatedly reported an association between UPF consumption and the risk of obesity [6–8], diabetes (both type 2 and gestational diabetes) [9, 10], cardiovascular diseases [11, 12], and other non-communicable diseases [13]. Moreover, recent prospective studies have showed an association between UPF consumption and some functional gastrointestinal disorders or diseases, such as irritable bowel syndrome, functional dyspepsia [14] and Crohn’s disease [15, 16]. However, the association between UPF consumption and peptic ulcer disease (PUD) has not been investigated.

PUD is characterized by an acid peptic lesion of the gastrointestinal mucosa, with depth to the submucosa. Ulcers are generally located in the stomach and proximal duodenum, but can sometimes affect the lower esophagus, distal duodenum or jejunum. The most common symptom of PUD is the burning epigastric pain occurring after meals. Gastrointestinal bleeding is the most common complication, with a mortality rate of 2.5–10%, mainly due to non-hemorrhagic causes such as multiorgan failure, pulmonary complications, and malignancy. Other PUD-related complications are perforation, with a mortality rate of 20%, and gastric outlet obstruction [17]. It has been estimated that 5–10% of individuals in the general population develop PUD during their lifetime. *Helicobacter pylori* infection and use of nonsteroidal anti-inflammatory drugs (NSAIDs) and aspirin are the most common risk factors for PUD. However, the pathophysiology of ulcers not associated with *H. pylori* or NSAID ingestion is becoming more relevant as the incidence of *H. pylori* is dropping, particularly in the Western world [17].

Diet has been thought to play a role in the development of PUD, but the evidence is limited and controversial. Certain diet components, such as salt, refined carbohydrates, alcohol, fiber, vitamins and polyphenols have been suspected to be linked to the PUD risk [18–20]. Since UPF are characterized by a low content of protective nutrients, such as fiber, vitamins and polyphenols, and a high density of sugars and salt, a diet rich in these products could contribute to the development of PUD. To elucidate this issue, we conducted an analysis in the SUN cohort to appraise whether UPF consumption was associated with the incident risk of PUD.

## Materials and methods

### Study design and participants

The SUN project is a Spanish dynamic prospective cohort aimed on studying the relationship between dietary habits, lifestyle and health status. The recruitment began in 1999 and it still on going. Participants are former graduates of University of Navarra, health professionals and other graduates [21]. Participants were invited to participate by means of a letter (e-mail or regular mail) outlining the objective of the project, what their participation entailed, the information required over time, and the arrangements put in place to safeguard their privacy. Along with the invitation letter, participants received a questionnaire (online or paper) designed to collect basic information on sociodemographic and lifestyle variables, eating habits, and medical history. They were given the option to decline participation in the study simply by not submitting the completed questionnaire. Therefore, voluntary completion of the first questionnaire was considered as informed consent as it was approved by the Ethical Committee. Every two years, participants received a new questionnaire investigating the occurrence of new diseases. Ten years after entering the cohort, the questionnaire sent to participants also investigated dietary habits so that they could be updated. This does not imply termination of the study. In fact, participants continue to receive the health status update questionnaire every two years. Participants who were recruited in 1999 have more than 20 years of follow-up within the SUN database used for this study. The project conformed to the guidelines established in the Declaration of Helsinki, and the Human Research Ethical Committee at the University of Navarra approved all the study procedures (091/2008).

**Fig. 1:**
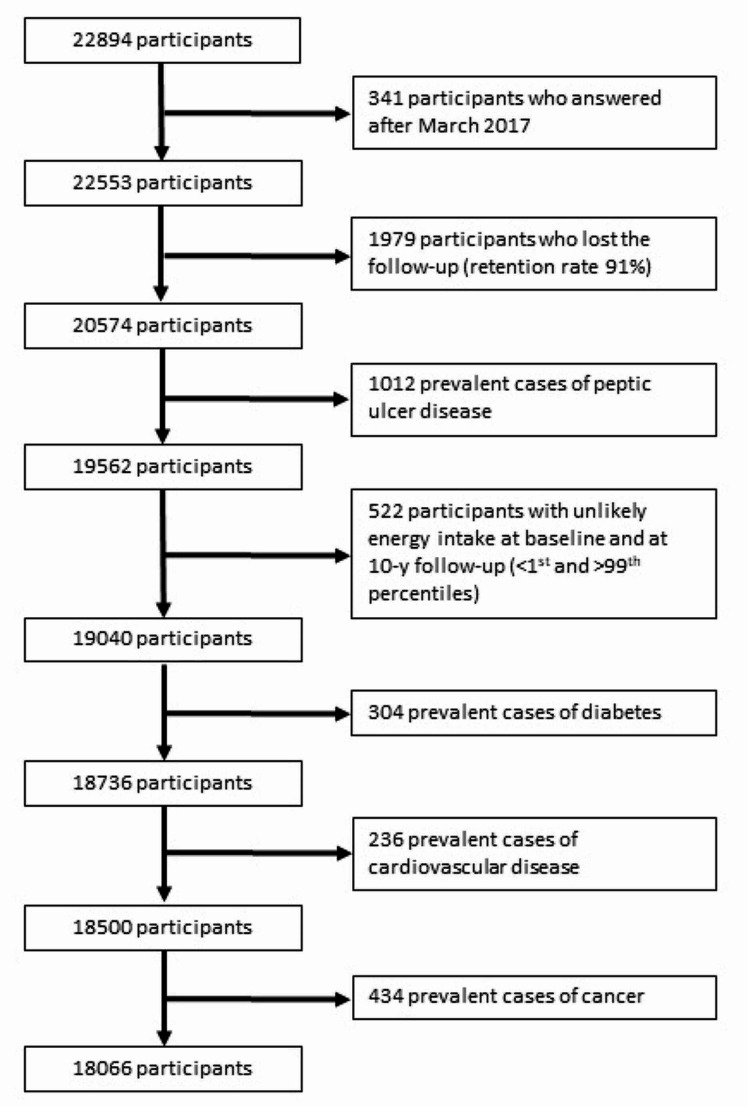
Flow chart showing the selection process of participants in the SUN project to be included in the present analysis

Up to December 2019, 22,894 participants completed the baseline questionnaire. To ensure two years in the cohort, 341 participants who responded the baseline questionnaire after March 2017 were excluded. We further excluded 1979 participants with no follow-up (retention rate 91%), 1012 participants with a history of PUD, 522 participants with unlikely energy intake (< 1st percentile and > 99th percentile), 304 participants with diabetes, 236 prevalent cases of cardiovascular disease, and 434 participants with a prior diagnosis of cancer. The final dataset included 18,066 participants who answered at least 1 follow-up questionnaire (Fig. 1).

### Exposure Assessment

We assessed dietary habits at baseline and again after 10 years using a validated semi-quantitative food frequency questionnaire consisting of 136-food items [22]. Each food item included a typical portion size. Participants were asked to report the frequency of consumption of each food item by selecting one of 9 frequency options reported in the questionnaire, ranged from never or almost never to more than 6 servings per day. We multiplied the frequency of consumption by the typical portion size to estimate the daily consumption of each food. Foods and beverages were then classified into one of the four categories of the NOVA classification [23]. The UPF group included processed meat, sausages, cookies and pastries, chocolate and candy, breakfast cereals, sweet or salty packaged snacks, margarine, instant soups, pre-prepared pies and pizza dishes, fruit yogurt, carbonated beverages, sweetened milk and fruit drinks, and alcoholic beverages produced by fermentation followed by distillation such as whiskey, gin and rum (a total of 34 items). To estimate the amount of UPF consumed daily, we summed the amount consumed (g/day) of each food item included in the UPF group. We then adjusted the consumption of UPF for the daily energy intake through the residuals method [24]. The use of daily grams of UPF instead of its caloric contribution made it possible to also consider foods that do not provide calories (e.g., calorie-free sweetened beverages). After that, we divided the sample into tertiles according to the total UPF consumption.

### Outcome Assessment

Participants reporting a medical diagnosis of PUD in one of the follow-up questionnaires were defined as incident cases of PUD. To validate the self-reported diagnosis of PUD, a subgroup of 139 participants from the SUN cohort was randomly selected (51 reporting PUD and 88 not reporting PUD), and the information reported was compared against the medical history in the clinical records available at the university clinic. A gastroenterologist, blinded to the exposure, handled the comparison. From those who reported a diagnosis of PUD (*n* = 51), 30 (58.8%; 95% confidence interval [CI], 44.2-72.4% were confirmed through their medical history. From the rest (*n* = 21), 19 (90.5%, 95% CI: 69.6-98.8%) were diagnosed as gastritis (*n* = 14), esophagitis (*n* = 18) and/or epigastric pain (*n* = 1), and only 2 they did not have any diagnostic related to gastric disease. From the 88 who did not report a diagnosis of PUD, all (100%; 95% CI: 88.4-100%) were confirmed as non-cases of PUD.

### Covariates

The baseline questionnaire collected information on sex, age, sociodemographic characteristics, weight and height, smoking, physical activity, and medical history. Self-reported weight and height were previously validated in a subgroup of our cohort [25]. Physical activity was assessed using the Spanish version of the 17-item Harvard Nurses’ Health Study physical activity questionnaire [26]. Leisure time activities were measured in metabolic activity equivalents (METs) per week by assigning habitual energy expenditure to each activity and multiplying by the time spent (in hours per week) on each activity. Total energy and nutrient intake were estimated from food consumption analyzed by the semi-quantitative FFQ using the most up-to-date version of the Food Composition Database for Spain.

### Statistical analysis

Continuous variables are reported as median and interquartile range (IQR) because some descriptive variables did not have a Gaussian distribution. Discrete variables are reported as count and percent. A Cox regression model, stratified by smoking and physical activity, was conducted to evaluate the association between tertiles of UPF consumption and PUD risk. The hazard ratio (HR) was calculated using the lowest tertile as the reference category. To control for possible confounders, sex, age (decades), BMI (quartiles), calendar year of recruitment (1999–2001, 2002–2004, 2005–2007, 2008–2010, and from 2011 onwards), health career (yes/no), education (3–4, 5–6, 9 years), marital status (unmarried, married, other, missing), packs of cigarettes (0, 1–12 packs/year, 13–24 packs/year, > 24 packs/year, missing), energy intake (quartiles), known *H. pylori infection* (yes/no), gastroesophageal reflux (yes/no), hiatal hernia (yes/no), aspirin use (yes/no), NSAIDs use (yes/no), coffee consumption (no, 1–2 cups/day, > 2 cups/day), and alcohol intake from wine and beer (quartiles) were included in the model. Confounders were selected based on biological plausibility and previous causal knowledge on the topic as it is recommended by Hernan et al. [27]. Although we adjusted for a large number of confounding factors, we cannot rule out residual confounding. UPF consumption could also be closely related to other aspects of an unhealthy lifestyle. To assess this aspect, we calculated Vanderweele’s proposed E value [28]. This value represents the minimum strength of association on the risk ratio scale that an unmeasured confounder would need to have with both the UPF consumption and the PUD to fully explain away a specific exposure-outcome association, conditional on the measured covariates. The existence of a linear trend between exposure and outcome was assessed by assigning the median value of each tertile and treating the new variable as continuous. To confirm the result, we also ran the Cox regression model by including UPF consumption as a continuous variable (g/day). The linearity of exposure was assessed by the fractional polynomials method. We used repeated measures of dietary intake in order to consider possible changes in the consumption of UPF between baseline and after 10 years of follow-up. We used generalized estimation equations with family binomial and link logit to compute the odds ratio (OR). Only baseline exposure was considered for participants who developed PUD before the 10-year follow-up and those who did not develop PUD but had been in the cohort for less than 10 years. We also represented Nelson-Aalen survival curves, adjusted for potential confounding variables by means of inverse probability weighting methods, to describe the incidence of PUD over time across tertiles of UPF consumption. Sensitivity analyses was also carried out by rerunning the models under different scenarios: (1) changing energy limits, (2) including participants with prevalent diabetes, CVD and cancer, (3) excluding participants taking NSAIDs and aspirin, (4) excluding participants with prevalent gastrointestinal disorders, (5) excluding participants with obesity, (6) excluding participants with an alcohol intake > 25 g/day if woman and > 50 g/day if man, (7) additionally adjusting for sodium intake, (8) additionally adjusting for the adherence to the Mediterranean diet assessed by Trichopoulou score [29]. A p value < 0.05 was considered statistically significant. Statistical analysis was performed by means of STATA program, version 12.0 (StataCorp LP).

## Results

A total of 18,066 participants (62.9% women) were included in the final dataset. The main characteristics of the participants are presented in Table 1. Median age at baseline was 35 years (IQR: 27; 45), and median daily consumption of UPF was 260 g/day (IQR: 187; 357), contributing to 28.9% of total energy intake (IQR: 21.0; 36.0). Table S1 shows the percentage contribution of UPF subgroups to the total UPF consumption.

**Table 1:** Baseline characteristics of participants according to UPF consumption (*n*=18,066)

	Tertiles of energy adjusted ultra-processed food consumption						
	First (≤212 g/day) (*n*=6022)		Second (212–318 g/day) (*n*=6022)		Third (>318 g/day) (*n*=6022)		
	*N*	%	*N*	%	*N*	%	*P* value^Ŧ^
Women	4004	66.5	3847	63.9	3506	58.2	<0.001
Married	3591	59.6	2849	47.3	2323	38.6	<0.001
Years of university							<0.001
3-4 years	2191	36.4	2017	33.5	1911	31.7	
5-6 years	3231	53.7	3411	56.6	3542	58.8	
9 years	600	10	594	9.9	569	9.4	
Health career	4138	68.7	3812	63.3	3646	60.5	<0.001
Smoking status							<0.001
Current	1128	18.7	1324	22	1545	25.7	
Former	1954	32.4	1640	27.2	1351	22.4	
Taking aspirin	220	3.7	182	3	208	3.5	0.147
Taking NSAID	468	7.8	498	8.3	540	9	0.058
Helicobacter pylori infection	26	0.4	40	0.7	28	0.5	0.159
Hiatal Hernia	20	0.3	20	0.3	23	0.4	0.866
Gastroesophageal reflux	13	0.2	5	0.1	5	0.1	0.062
	**Median**	**IQR**	**Median**	**IQR**	**Median**	**IQR**	P value*
Age (years)	41	31; 50	34	27; 44	31	26; 39	<0.001
BMI (kg/m^2^)	22.9	20.8; 25.3	22.8	20.7; 25.3	22.9	20.8; 25.5	0.268
Physical activity (METs/week)	17.6	6.7; 32.4	15.2	5.6; 28.9	15.0	4.3; 30.0	<0.001
Energy (kcal/day)	2549	2122; 3071	2220	1802; 2718	2452	1996; 3025	<0.001
Macronutrients intake (% energy)							
Carbohydrate	44.5	39.4; 49.4	42.8	38.3; 47.2	43.4	38.9; 47.7	<0.001
Protein	17.9	16.0; 20.1	18.1	16.2; 20.2	17.2	15.4; 19.2	<0.001
Lipid	35.5	30.9; 39.9	37.0	33.2; 40.9	37.5	33.6; 41.1	<0.001
SFA	11.4	9.4; 13.4	12.6	10.8; 14.5	13.1	11.3; 15.0	<0.001
MUFA	15.3	12.9; 18.0	15.5	13.5; 17.7	15.4	13.6; 17.5	0.030
PUFA	4.7	3.9; 5.6	5.0	4.2; 6.1	5.3	4.3; 6.4	<0.001
Alcohol	0.8	0.2; 2.4	0.9	0.3; 2.5	0.9	0.2; 2.5	<0.001
Alcohol consumption (g/day)	2.7	0.6; 8.8	2.8	1.0; 8.1	3.3	1.0; 9.1	<0.001
Fiber (g/day)	32.8	25.5; 43.3	24.6	19.1; 31.8	23.7	17.9; 31.2	<0.001
Micronutrients intake							
Sodium (mg/day)	2771	2097; 3660	2763	2059; 3766	3523	2503; 4952	<0.001
Vit. A (μg/day)	2213	1408; 3253	1495	1065; 2370	1364	956; 2214	<0.001
Vit. C (mg/day)	324	227; 444	238	169; 332	224	158; 318	<0.001
Vit. E (μg/day)	7.1	5.3; 10.1	5.9	4.4; 8.2	6.3	4.7; 8.7	<0.001
Food consumption (servings/day)							
Olive oil (g/day)	25.0	10.7; 29.5	12.3	8.7; 25.8	11.8	8.0; 25.2	<0.001
Vegetables	2.6	1.8; 3.6	1.9	1.3; 2.8	1.8	1.2; 2.6	<0.001
Fruit	2.8	1.7; 4.3	1.8	1.1; 2.8	1.6	0.9; 2.6	<0.001
Red meat	0.5	0.3; 0.7	0.5	0.3; 0.7	0.5	0.3; 0.7	0.002
Processed meat	1.7	1.2; 2.3	1.8	1.3; 2.3	2.0	1.5; 2.7	<0.001
Sugar sweetened beverages	0.1	0.0; 0.1	0.1	0.1; 0.3	0.4	0.1; 0.9	<0.001
High-fat dairy products	1.4	0.6; 2.6	1.3	0.7; 2.2	1.5	0.8; 2.4	<0.001
Coffee consumption	1	0; 3	1	0; 3	1	0; 3	<0.001
Ultraprocessed food (servings/day)	2.5	1.9; 3.2	3.7	3.2; 4.3	4.9	4.2; 6.0	<0.001
Ultraprocessed food (g/day)	157.0	108.8; 186.9	259.8	235.4; 286.7	410.6	357.3; 506.5	<0.001
Ultraprocessed food/energy (%)	20.3	14.9; 25.7	29.2	23.8; 34.8	36.5	30.0; 43.2	<0.001

*Abbreviations* IQR, interquartile range; SFA, saturated fatty acids; MUFA, monounsaturated fatty acids, PUFA, polyunsaturated fatty acids; NSAID, nonsteroidal anti-inflammatory drugs

^Ŧ^ Chi-square test

* Kruskal Wallis test

During a median follow-up of 12.2 years, we recorded 322 new cases of PUD (1.56 cases/1000 person-years). The association between tertiles of UPF consumption and risk of PUD is reported in Table 2. Participants in the highest tertile of UPF consumption had a 52% increased relative risk of PUD compared to participants in the lowest tertile (HR = 1.52, 95%CI: 1.15, 2.00), with a significant dose-response relationship (P_trend_=0.002). A linear association between the UPF consumption and the occurrence of PUD was also confirmed when we included in the Cox regression model the exposure as a continuous variable. We observed a 7% increase in relative risk for every 100 g/day of UPF consumed (HR = 1.07, 95% CI: 1.03, 1.12). Furthermore, when we accounted for changes in UPF consumption (repeated-measures analysis), using the updated data on food consumption after 10 years of follow-up, the association remained statistically significant. Compared with participants in the lowest tertile, those in the highest tertile had a 39% increased risk of PUD (OR = 1.39, 95% CI: 1.03, 1.87; P_trend_ = 0.001).

**Table 2:** Association between consumption of ultra-processed foods and risk of peptic ulcer

	Tertiles of energy-adjusted UPF consumption					
	First (≤212 g/day)	Second (212–318 g/day)	Third (>318 g/day)	*P* for trend	HR (95% CI)	*P* value
No. of cases/no. of person-y	102/69,206	94/69,414	126/68,218			
No. of cases/no. of participants	102/6022	94/6022	126/6022			
Crude model	ref.	1.01 (0.76; 1.35)	1.47 (1.12; 1.92)	0.003		
Multivariable model	ref.	1.09 (0.81; 1.47)	1.52 (1.15; 2.00)	0.002		
Multivariable model (without BMI)	ref.	1.10 (0.82; 1.47)	1.51 (1.15; 2.00)	0.002		
Multivariable model (without energy intake)	ref.	1.05 (0.78; 1.40)	1.49 (1.13; 1.96)	0.003		
Multivariable model (linear, 100 g/day)					1.07 (1.03; 1.12)	0.001
Repeated measure multivariable model	ref.	1.07 (0.79; 1.47)	1.39 (1.03; 1.87)	0.001		

Multivariable model: adjusted for sex, age (decades), bmi (quartiles), calendar year of recruitment (1999–2001, 2002–2004, 2005–2007, 2008–2010, and from 2011 onwards), health career (yes/no), education (3-4, 5-6, 9 years), marital status (unmarried, married, other, missing), packs of cigarettes (0, 1-12 packs/year, 13-24 packs/year, >24 packs/year, missing), energy intake (quartiles), known Helicobacter pylori infection (yes/no), gastroesophageal reflux (yes/no), hiatal hernia (yes/no), aspirin (yes/no), analgesics (yes/no), coffee (no, 1-2 cups/day, >2 cups/day), alcohol from wine and beer (quartile), and stratified for smoking (categories) and physical activity (quartiles)

The observed HR of 1.52 in our analysis could hypothetically be explained by the presence of an unmeasured confounder associated with UPF consumption and PUD with a HR of 1.78-fold each, beyond the measured confounders, but a weaker confounder could not. Similarly, the lowest confidence interval could be moved to include the null by an unmeasured confounder associated with both UPF consumption and PUD by a HR of 1.36-fold each, above and beyond the measured confounders, but weaker confounding could not do so.

**Fig. 2:**
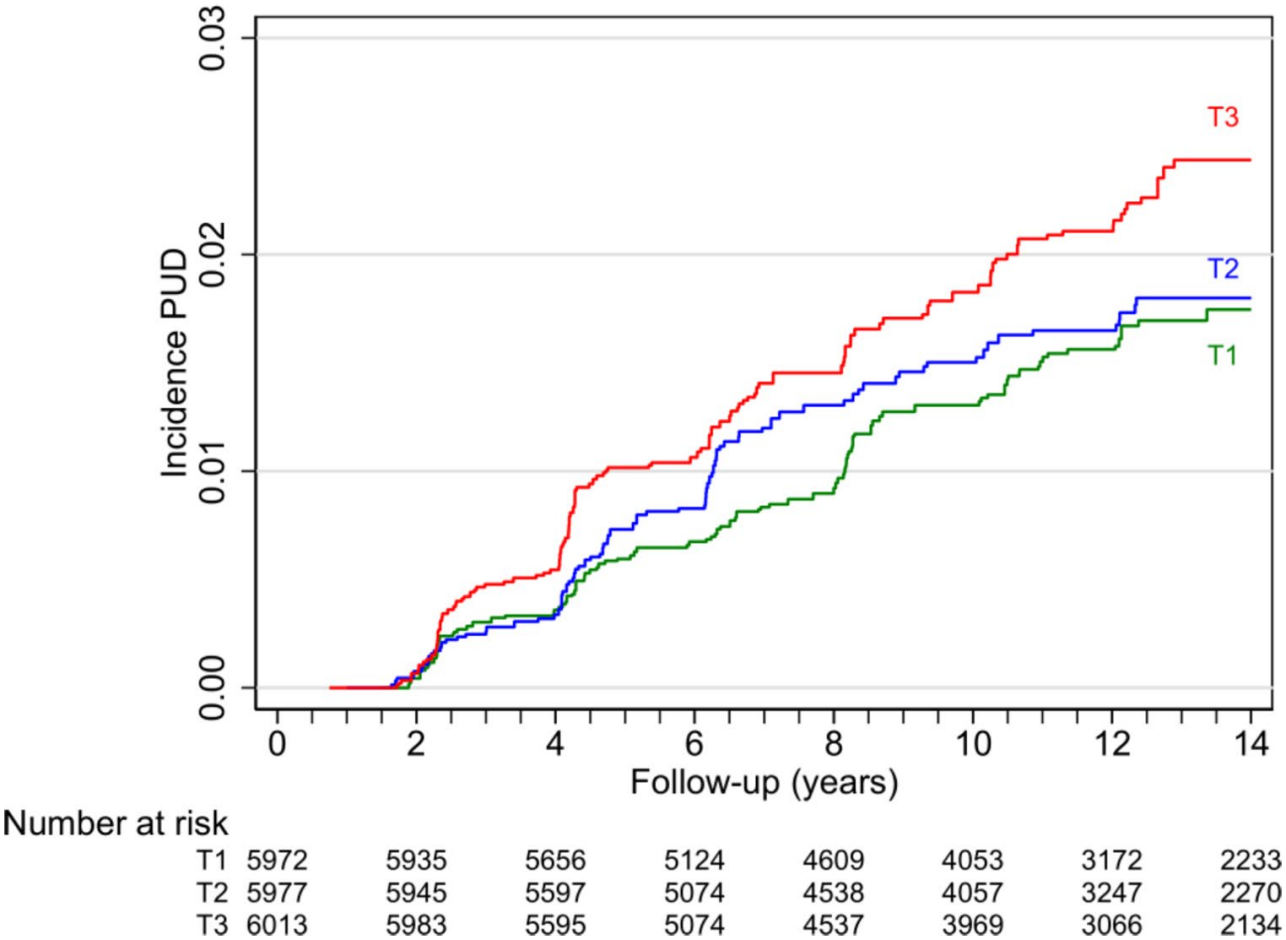
Nelson-Aalen estimate of the incidence of peptic ulcer disease according to tertiles of ultra-processed consumption

Figure 2 shows the cumulative risk for developing PUD over time across tertiles of UPF consumption. The highest tertile of UPF consumption was associated with higher incidence of PUD.

We also performed a sensitivity analysis to test the robustness of our results, but we did not observe any substantial change in the magnitude of the association in any of the examined scenarios (Table 3).

**Table 3:** Sensitivity analysis

		Tertiles of energy-adjusted UPF consumption			
	No. of cases/no. of person-y	T1	T2	T3	*P* for trend
Overall results	322/206,837	1 (ref.)	1.09 (0.81; 1.47)	1.52 (1.15; 2.00)	0.002
Willett’s energy limits (<800 kcal/d or >4000 kcal/d in men and <500 kcal/d or >3500 kcal/d in women)	298/192,450	1 (ref.)	0.99 (0.73; 1.35)	1.44 (1.08; 1.91)	0.008
Including participants with prevalent diabetes, CVD or cancer	341/217,437	1 (ref.)	1.06 (0.80; 1.41)	1.47 (1.17; 1.92)	0.003
Excluding participants taking aspirin and analgesics	271/183,571	1 (ref.)	1.09 (0.79; 1.50)	1.57 (1.17; 2.13)	0.003
Excluding participants with prevalent gastrointestinal disorders	316/204,812	1 (ref.)	1.07 (0.80; 1.45)	1.48 (1.12; 1.95)	0.004
Excluding participants with obesity	303/198,598	1 (ref.)	1.12 (0.82; 1.52)	1.61 (1.21; 2.15)	0.001
Excluding participants with alcohol consumption >25 g/d for women or >50 g/d for men	314/203,550	1 (ref.)	1.12 (0.83; 1.50)	1.51 (1.14; 2.00)	0.003
Additionally adjusted for sodium intake	322/206,837	1 (ref.)	1.10 (0.82; 1.48)	1.55 (1.16; 2.06)	0.002
Additionally adjusted for Mediterranean diet	322/206,837	1 (ref.)	1.08 (0.80; 1.46)	1.50 (1.13; 1.99)	0.003

Multivariable model: adjusted for sex, age (decades), bmi (quartiles), calendar year of recruitment (1999–2001, 2002–2004, 2005–2007, 2008–2010, and from 2011 onwards), health career (yes/no), education (3-4, 5-6, 9 years), marital status (unmarried, married, other, missing), packs of cigarettes (0, 1-12 packs/year, 13-24 packs/year, >24 packs/year, missing), energy intake (quartiles), Helicobacter infection (yes/no), gastroesophageal reflux (yes/no), hiatal hernia (yes/no), aspirin (yes/no), analgesics (yes/no), coffee (no, 1-2 cups/day, >2 cups/day), alcohol from wine and beer (quartile), and stratified for smoking (categories) and physical activity (quartiles)

## Discussion

In this prospective cohort study, we found that a higher consumption of UPF was associated with the risk of incident PUD. This result remained consistent in the sensitivity analysis even when excluding participants taking NSAIDs and aspirin and with gastrointestinal disorders, including a known *H. pylori* infection. However, it should be kept in mind that *H. pylori* infection is often asymptomatic. Therefore, it is highly likely that many participants did not know they were infected. The further adjustment for the adherence to the Mediterranean diet, dietary pattern rich in unsaturated fatty acids, fiber, vitamins and minerals and antioxidants, and low in salt, did not affect the result. Thus, our findings support the hypothesis that the consumption of UPF could be an environmental factor that increases the risk of PUD. Recent systematic reviews and meta-analyses reported an increased risk of functional gastrointestinal disorders or diseases, including dyspepsia, irritable bowel syndrome [13], and Crohn’s disease [30], associated with higher consumption of UPF. Furthermore, a recent case-control study showed the consumption of UPF associated with a higher risk of stomach adenocarcinoma [31]. However, to the best of our knowledge, this is the first study investigating the association between UPF consumption and PUD.

Several mechanisms could explain the relationship between UPF and PUD. Recent meta-analysis reported that a higher UPF consumption contributed to increase dietary intake of salt and refined carbohydrates, and to reduce the intakes of fiber, vitamins, and antioxidants [32]. Our data confirmed these findings, also showing higher alcohol intake among those who consumed higher amounts of UPF, presumably due to higher consumption of distilled spirits and liquor (alcoholic beverages that fall under the definition of UPF). Epidemiological studies have reported these components in the diet to be positive or negative associated with PUD risk. In addition, results from animal studies have corroborated these findings, providing evidence on possible mechanisms. In particular, some diet components may undermine the integrity of the mucosal barrier, resulting in inflammation and damage, and subsequently, erosion of the gastric mucosa. The mucosal damage could enhance *H. pylori* colonization and the presence of certain nutrients in the gastric lumen may influence the expression of *H. pylori* virulence factors associated with the development of PUD and other gastroduodenal diseases [33]. In vivo studies shown salt to alter the viscosity and composition of the protective mucosal barrier [34, 35], potentially exposing the mucosa to the toxic effects of acid and intragastric enzymes, resulting in mucosal damage and inflammation [36]. Moreover, salt has been also shown to facilitate *H. pylori* colonization both in human and animals [37–39] and increase gene expression of virulence factors which resulted in more virulent bacterial strains [40]. Prospective and geographical studies have confirmed the role of salt, documenting an increased risk of gastric ulcer associated with higher salt intake [20, 41]. Other prospective studies reported the intakes of refined carbohydrates and fiber, especially soluble fiber from fruits, vegetables, and legumes, associated with higher and lower risk of duodenal ulcer, respectively [18, 19]. These findings were corroborated by additional studies showing an increased risk of *H. pylori* infection associated with carbohydrate/sugar intake and diet glycemic index [42, 43]. Moreover, in patients with duodenal ulcer, it has been observed that the liquid phase of a meal is emptied more rapidly into the duodenum, compared with controls [35]. A rapid rate of gastric emptying in the presence of gastric hypersecretion may play an important role in the pathogenesis of duodenal ulcer. Dietary fiber might delay the gastric emptying explaining its apparent benefit. The relationship between alcohol intake and PUD risk is uncertain. However, alcohol is known to dose-dependently damage the gastric mucosa through numerous mechanisms, including alterations in epithelial transport, disruption of the intercellular junction and mucosal barrier, which allow hydrogen ions to penetrate the mucosa [44]. Moreover, ethanol activates an inflammatory reaction that also participates in gastric mucosal damage [45]. Histological studies indicate that after ethanol administration in concentrations comparable to distillates and spirits (20% and 40% ethanol), the mucosal layer and mucin content of the lining epithelial cells decreased significantly. In addition, the presence of acids aggravated the injury and induced bleeding. The restoration of mucosal damage was completed in 24 h [46]. Alcoholic beverages such as wine and beer may be less harmful to the gastroduodenal mucosa both for lower ethanol content and for the presence of polyphenols, which have shown anti-ulcer effects such as reducing acid secretion, inhibiting pepsin level and activity, and increasing gastric mucus and bicarbonate secretion, as well as enhancing cytoprotective, antioxidative, anti-inflammatory, and antibacterial mucosal defenses against peptic ulcer [47]. Finally, it has been suggested that some vitamins, whose intakes were lower in participants consuming larger amounts of UPF, may protect against PUD through several mechanisms. In animal models, vitamin A increased gastric and duodenal mucus production. Moreover, dietary supplementation of vitamin A reduced the incidence of stress- and aspirin-induced ulcers in rats [48]. Vitamin E, particularly tocopherol and tocotrienol, conferred its protection against ulcerogenic factors/agents mainly through its antioxidant and anti-inflammatory mechanisms [49]. In addition, vitamin C attenuated the oxidative damage induced by NSAIDs and *H. pylori* to the gastric mucosa [50, 51]. Note, however, that so far only vitamin A intake has been found to be associated with a lower risk of duodenal ulcer in humans [18].

We are well aware that our study is not free of limitations. First, the ulcer diagnosis was self-reported and validation study showed partial validity. Therefore, we have to acknowledge the existence of some misclassification, taking in mind that some of self-reported diagnoses of PUD are gastritis or esophagitis. Nevertheless, the plausible biological mechanisms explained before can be applied as well. Second, we had no information on ulcer location and therefore could not investigate the impact of UPF on gastric and duodenal ulcer risk separately. Third, we had no information on the use of proton pump inhibitors that may have influenced the risk of PUD. However, we controlled the analysis for gastrointestinal disorders, like hiatal hernia and gastroesophageal reflux, that generally require the use of proton pump inhibitors. Fourth, like any FFQ, the one used in this study has the limitation of investigating the consumption of only the foods listed. We are aware that consumption was not specifically required for all commercially available UPF. This may have led to an underestimation of exposure. Nevertheless, this is a validated FFQ investigating a large number of food items (*n* = 136). Fifth, SUN cohort involves mainly graduate participants, limiting the generalizability of our results. Sixth, since this was a cohort of Spanish graduates, we can assume that almost all participants were Caucasian, and therefore these results cannot be transferred to other ethnicities without prior confirmation. Finally, as in any observational study, we cannot rule out residual confounding. However, adjusted for a wide range of potential confounders using different statistical methods, and the results were consistent. In addition, the E-values for the point estimate supported the observed association. The point estimate could be theoretically explained only by an unmeasured confounder with a hazard ratio of at least 1.78-fold for PUD and for ultra-processed food consumption.

Our study has several strengths. First, the study addresses a topic not previously covered. Second, the study prospective nature, as the assessment of participants’ dietary habits was carried out before the onset of the disease, which reflects the optimal temporal relation between exposure and disease occurrence. Third, the repeated measurements of exposure variable allowed to take into consideration dietary changes occurred overtime. Fourth, we controlled our analysis for NSAID and aspirin use and for the presence of gastroduodenal disorders, including known *H. pylori* infection, risk factors for PUD. However, as mentioned earlier, it is likely that many participants did not know they were infected due to lack of symptoms. Therefore, it is not possible to extrapolate detailed information about the underlying mechanisms. Fifth, the high cohort response rate. Sixth, the high educational level of participants, which may have facilitated better understanding of the food frequency questionnaire. Seventh, the food frequency questionnaire used to assess dietary habits has been repeatedly validated [52, 53].

In conclusion, our data suggest that the consumption of UPF may be associated with the risk of PUD and other gastric disorders. Given the importance of the topic - individuals with PUD have an increased risk of developing gastric cancer [54] - further studies confirming our findings are strongly requested. Nevertheless, UPF consumption should still be discouraged because of the known negative associations with health status.

## Electronic supplementary material

Below is the link to the electronic supplementary material.

Supplementary Material 1
