# Supplementary material for: Association between the consumption of ultra-processed foods and the incidence of peptic ulcer disease in the SUN project: a Spanish prospective cohort study
Source: Eur J Nutr. 2024 May 29;63(6):2367–78. doi: 10.1007/s00394-024-03439-2 (PMC11377682; doi:10.1007/s00394-024-03439-2)
Supplement: Supplementary file 1 — Supplementary Material 1 [file 394_2024_3439_MOESM1_ESM.docx]

Table S1: Contribution of UPF subgroups to UPF consumption in SUN cohort

|  | **Median** | **P25** | **P75** | **mean** | **sd** |
| --- | --- | --- | --- | --- | --- |
| Sugar-sweetened and sugar-free beverages (%) | 19.3 | 6.7 | 38.9 | 24.7 | 22.2 |
| Cookies, sweets, chocolates, desserts (%) | 15.9 | 8.4 | 26.8 | 19.7 | 15.5 |
| Ultraprocessed meat products (%) | 13.7 | 8.1 | 21.6 | 16.3 | 11.9 |
| Margarine and fried products (e.g. french fries) (%) | 8.1 | 3.7 | 15.8 | 11.5 | 11.5 |
| Miscellaneous (e.g. sauces, instant soups, nuggets, sweeteners) (%) | 8.1 | 3.9 | 15.1 | 11.4 | 11.5 |
| Ultraprocessed dairy products (%) | 7.1 | 2.9 | 13.2 | 9.7 | 10.2 |
| Ultraprocessed cereals (e.g. breakfast cereals, pizza) (%) | 2.4 | 0.9 | 6.0 | 5.3 | 8.5 |
| Distillates and spirits (%) | 0.0 | 0.0 | 1.6 | 1.5 | 3.7 |

Abbreviations: P25, 25^th^ percentile; P75, 75^th^ percentile
